# Food insecurity, school absenteeism and educational attainment of adolescents in Jimma Zone Southwest Ethiopia: a longitudinal study

**DOI:** 10.1186/1475-2891-10-29

**Published:** 2011-04-10

**Authors:** Tefera Belachew, Craig Hadley, David Lindstrom, Abebe Gebremariam, Carl Lachat, Patrick Kolsteren

**Affiliations:** 1 Department of Population and Family Health, College of Public Health and Medical Sciences, Jimma University, PO.Box:1104, Jimma, Ethiopia; 2 Department of Food Safety and Food Quality, Faculty of Bioscience Engineering, Ghent University, Coupure links 653, 9000 Gent, Belgium; 3 Department of Anthropology, Emory University, 207 Anthropology Building 1557 Dickey Drive, USA; 4 Department of Sociology, Brown University, Box 1916, Providence, RI 02912, USA; 5 Nutrition and Child Health Unit, Department of Public Health, Institute of Tropical Medicine, Nationalestraat 155, 2000 Antwerpen, Belgium

## Abstract

**Background:**

Food insecurity not only affects physical growth and health of children but also their intellectual development, school attendance and academic performance. However, most evidences are based on studies in high income countries. Although food insecurity is common in Ethiopia, to what extent it affects school attendance and educational attainment of adolescents is not explored. We hypothesized that food insecure adolescents would be more likely to be absent from school and have lower grades attained after 1 year compared to their food secure peers.

**Methods:**

We used data from 2009 adolescents in the age group of 13-17 years from two consecutive surveys of a five year longitudinal family study in Southwest Ethiopia. A stratified random sampling was used to select participants. Regression analyses were used to compare school absenteeism and the highest grade attained after 1 year of follow-up in food secure and insecure adolescents. The analysis was adjusted for demographic factors, reported illness and workload.

**Results:**

Significantly more (33.0%) food insecure adolescents were absent from school compared with their food secure peers (17.8%, P < 0.001). Multivariable logistic regression analyses showed that after adjusting for gender, place of residence and gender of the household head, adolescent food insecurity [OR 1.77 (1.34-2.33)], severe household food insecurity [OR 1.62 (1.27-2.06)], illness during the past one month before the survey [OR 2.26 (1.68-3.06)], the highest grade aspired to be completed by the adolescent [OR 0.92 (0.88-0.96)], and the number of days that the adolescent had to work per week [OR 1.16 (1.07-1.26)] were independent predictors of school absenteeism. Similarly after controlling for household income and gender of the household head, adolescent food insecurity(P < 0.001), severe household food insecurity(P < 0.001), illness during the last month(P < 0.001) and rural residence(P < 0.001) were inversely associated with highest grade attained, while age of the adolescent(P < 0.001), the highest grade intended to be completed(P < 0.001) and residence in semi urban area(P < 0.001) were positively associated with the highest grade attained.

**Conclusions:**

Adolescent and household food insecurity are positively associated with school absenteeism and a lower educational attainment. Programs aiming to achieve universal access to primary education in food insecure environments should integrate interventions to ensure food security of adolescents.

## Background

Optimal cognitive and emotional development and physiological function in children and adolescents requires access to food of adequate quantity and quality at all stages in life. Recurrent food insecurity as experienced in Ethiopia [1] may result in malnutrition with resulting developmental impairments such as poor learning capacity in children [2-4]. Food insecurity adversely affects intellectual development [2,3,5-7], school attendance [8], growth [7], health [9] academic performance and social skills [2,5,7] of children and adolescents.

There are different mechanisms through which food insecurity could negatively affect educational attainment. A frequently mentioned pathway is through high rates of school absenteeism, with children living in food insecure households being pulled form school to engage in productive activities. It has been hypothesized that this might be particularly true for girls. Another of the mechanisms is through decreased intake of nutrients during periods of reduced food supply. Although various studies have shown that adult household members buffer children from food insecurity [10-13]; alterations in the eating behaviour of food insecure adolescents in a way that affects their nutritional status [8,14] have been reported. Children who experience food insecurity might also suffer from a significant amount of psychological and emotional stress at home around the concerns of the caregivers to provide adequate food. This may affect the emotional well-being of the adolescent to an extent that interferes with cognitive [15-17] and behavioral performance [18]. Food-insufficient teenagers were reported to have behavioral problems that are incompatible with their school attendance [5].

Several longitudinal [2,3,19] and cross-sectional [5,6,18] studies in high income countries have shown a relationship between household food insecurity as reported by the head of the household and adverse educational outcomes in younger children. This relationship, however, has not been explored in low income countries. To our knowledge there are no studies on the effect of adolescent's personal experience of food insecurity on school absenteeism and educational attainment from both high or from low income countries where food insecurity is prevalent. Assessing the link between adolescent food insecurity and school absenteeism and educational attainment is critical to design and integrate multi-sectoral strategies aiming to achieve the Millennium Development Goal 2, "Universal Access to Primary Education", in Ethiopia. We hypothesized that the proportion of school absentees would be higher in the food insecure adolescents compared to their food secure peers and that the food insecure adolescents would have a lower educational attainment as well.

## Subjects and methods

### Study sample

This study is based on data from 2009 adolescents enrolled in the first two consecutive rounds of the five year longitudinal study of adolescents in Jimma zone in Southwest Ethiopia. The study area was stratified into urban (Jimma city), semi urban (small towns) and six rural communities "kebeles" adjacent to the towns, representing a range of ecological and developmental contexts. A census generated a list of all 5795 households in the study sites. A two-stage sampling plan was used to select a representative sample of 2100 adolescents from this list. First, 3700 households were selected from this list based on a probability proportional to size of the household. Next, one adolescent (a boy or a girl) was selected from each household using a Kish table [20].

### Measurements

Structured questionnaires for the households and adolescents were used to collect data. The interviewers received one week of intensive training prior to the pre-test and an additional week of training was given with the final version of the questionnaire before the start of the actual interviews. Supervisors checked the data collection process and filled questionnaires daily to ensure accuracy of the data. The research team supervised the data collection team every week through meetings and checking of the filled questionnaires. The first round of data collection was carried out from mid - 2005 to 2006, while the second round was finalized around same time in 2006-2007. The timing of both rounds corresponds to the rainy season (hunger season) and spring season which is relatively better in terms of food security.

Food insecurity is a situation that exists when all people, at all times, do not have physical, social and economic access to sufficient, safe and nutritious food that meets their dietary needs and food preferences for an active and healthy life [21]. Both adolescent and household food insecurity were measured in the first round of the survey using items from previously adapted and validated household food insecurity scales for use in the developing countries [22-24]. Adolescent food insecurity was measured with a modified version of the household food security scales by selecting the items in the scales that apply to their personal experiences. Adolescents were asked to think of their own experience and not that of the household and then asked whether in the last three months they had (1) ever worried about having enough food (2) ever had to reduce food intake because of shortage of food or lack of money to buy food (3) ever had to go without eating because of shortage of food or money to buy food (4) ever had to ask outside the home for food. All "Yes" responses were coded as one and "No" responses were coded as zero, and the responses were summed to produce an index of adolescent food insecurity. The index had an adequate internal consistency (Cronbach's alpha = 0.81) and was further dichotomized as "food secure" for a score equal to zero "food insecure" for a score is greater than zero.

To assess household food insecurity, a series of six questions were presented to the household head. The questions asked whether during the past three months (1) the respondent worried about running out of food (2) the household ran out of food (3) the variety of food for children was reduced (4) the children did not have enough to eat (5), the respondent or another adult did not have enough to eat (6) and the respondent ever felt hungry but did not eat. The "Yes" responses were coded one and the "No" responses were coded zero, and the responses were summed to produce an index of household food insecurity. The index had a high internal consistency (Cronbach's alpha = 0.92). The distribution of the food insecurity index was divided into tertiles and the highest tertile was labeled as "severely food insecure" in contrast to the two lower tertiles combined which were coded zero. Severe household food insecurity was used in subsequent analyses as we previously documented that adolescents in the region suffer more from food insecurity when household food insecurity is severe [13].

The questionnaires were translated to the local languages (Amharic and Oromifa) and the administered by an interviewer. The consistency of the forms was checked by a researcher other than the interviewers.

During the first round of the survey, school absenteeism and other socio-demographic variables were recorded. School absenteeism was defined as "any illegitimate absence from school for at least a day" [25,26]. An illegitimate absence was defined as any absence from school due to reasons others than the formal school closure days (due to either national holidays or religious days for which the school is closed). Adolescents were asked: "Thinking back to the full semester you were in school, how often did you miss school?" The possible responses were: "several days a week", "several days a month", "several days in the semester" and "never". We coded the responses "several days a week", "several days a month" and "several days in the semester" as absentees and "never" as non-absentees.

The educational attainment was assessed one year later by asking the adolescents, "What is the highest grade you have completed?" The questionnaire was tested on 200 adolescents (not included in the sample) selected from a community in Jimma city.

The study was cleared by the Ethical Review Boards of both Brown University (USA) and Jimma University (Ethiopia). Informed verbal consent was obtained both from the parents and each adolescent before the interview.

### Data analysis

The data were entered in double, checked for missing values and outliers and analyzed using SPSS for windows version 16.0 (SPSS Inc. version 16.1, Chicago, Illinois). First, bivariate analyses were carried out. Means and proportions were compared by school absenteeism using t-test and Chi-square tests, respectively. To identify the predictors of school absenteeism, a multivariable logistic regression model with school absenteeism as dependent variable was constructed. Variables that showed a significant association with school absenteeism in the bivariate analyses were entered in a multivariable logistic model. Interaction between variables was checked at the level of significance of P < 0.05. A second multivariable linear regression model was fitted to assess factors predicting educational attainment. All variables that were significantly associated (P < 0.05) with the highest grade completed in the bivariate models were entered into the adjusted linear model to assess their independent effects. Normality of the data was assessed visually using a P-P plot for all numerical predictor variables and for the dependent variable (highest grade completed) in the regression model. All variables were normally distributed and there was no need of transformation. Co-linearity between household food insecurity and adolescent food insecurity were checked using correlation coefficients and variance inflation factor test. All tests were two-sided and a P < 0.05 was considered statistically significant. We present the results as means ± SD, output of the logistic regression as odds ratios (OR) with 95% confidence intervals and the results from the linear regression model as β-coefficients and P values.

## Results

Out of the 2100 adolescents intended, 2084 completed the questionnaires. For further analysis of absenteeism we excluded 75 adolescents who were not enrolled in the school. Bivariate analyses showed that a significantly higher proportion (33.0%) of food insecure adolescents was identified as absentees compared with food secure youth (17.8%), P < 0.001. School absenteeism was positively associated with household food insecurity (P < 0.001) and number of hours per week that the adolescents had to work for the household (P < 0.001). Similarly, 51.0% adolescents from households with severe food insecurity reported absenteeism compared to 36.3% of adolescents who were not part of severely food insecure households (P < 0.001). A larger proportion (24.9%) of adolescents who reported an illness during one month prior to the survey also reported absenteeism compared to those who did not report an illness (11.1%, P < 0.001). The mean educational level attained (Table 1), expressed as the highest grade completed at the second survey was 6.2 ± 2.5 for non-absentees and 5.9 ± 2.3, for absentees (P = 0.047).

**Table 1:** Characteristics of adolescents in Southwest Ethiopia by school absenteeism

Characteristics	Non-absentees [n 1660]	Absentees [n 349]	P
Gender			
Male	51.8%	46.4%	0.067
Female	48.2%	53.6%	
Age (Years) †	14.8 (1.3)	14.7 (1.3)	0.277
Residence*			
Urban	36.9%	36.7%	0.216
Semi-urban	28.5%	32.7%	
Rural	34.6%	30.7%	
Adolescent food insecurity			
Food secure	82.1%	67.0%	< 0.001
Food insecure	17.8%	33.0%	
Severe household food insecurity			
No	63.7%	49.0%	< 0.001
Yes	36.3%	51.0%	
Illness during the last one month before the survey			
Yes	88.9%	75.1%	< 0.001
No	11.1%	24.9%	
Being part of female headed household			
No	82.4%	80.5%	0.402
Yes	17.6%	19.5%	
Number of days in a week the adolescent has to work for at least 1 hour†	1.9(1.6)	2.3(1.3)	< 0.001
The highest grade the adolescent aspires to complete†	13.7(6.9)	13.3(4.9)	0.006
The highest grade attained at round 2 of the survey†	6.2(2.5)	5.9(2.3)	0.047

*"Semi-urban" refers to small towns including Yebbu, Serbo and Dedo and "urban" refers to Jimma City.

Means and proportions were compared using t-test and Chi-square tests, respectively.

^†^Means with standard deviations in brackets are shown, unless otherwise indicated.

Percentages are computed out of row totals.

**Figure 1:**
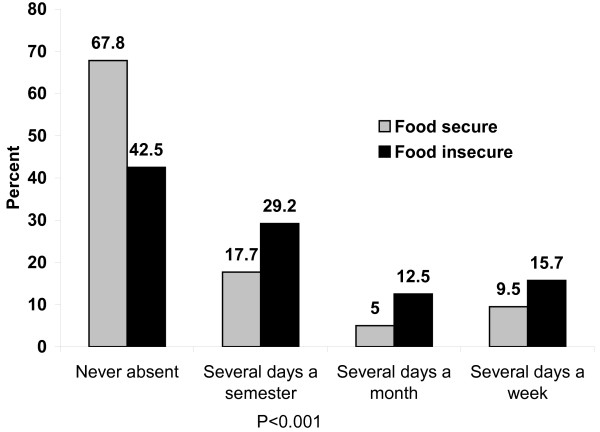
**Frequency of school absenteeism by food security status of adolescents in Jimma Zone Southwest Ethiopia**.

**Figure 2:**
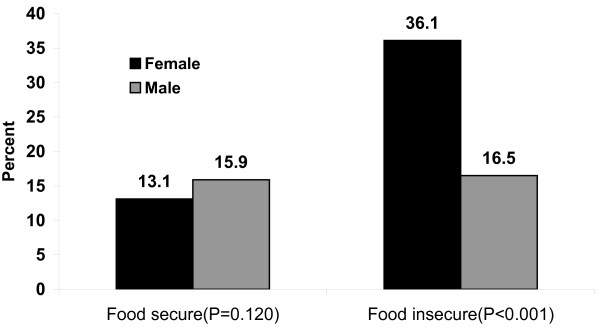
**School absenteeism by food security status and gender of adolescents in Southwest Ethiopia**.

Figure 1 shows the frequency of school absenteeism by food security status of adolescents. The frequency of school absenteeism was significantly higher among food insecure adolescents (P < 0.001). While the majority (67.8%) of food secure adolescents was never absent, the frequency of school absenteeism among food insecure youth ranged from several days per week (29.2%) to several days per semester (15.7%). After stratification by gender, a significantly (P < 0.001) higher proportion (34.5%) of food insecure girls were absent compared to boys (15.0%). This gender difference in absenteeism was not significant(P > 0.05) in the food secure adolescents (Figure 2).

The mean (± SD) highest grade completed after 1 year was 6.0(± 2.7) for food secure adolescents and 5.5(± 2.7) for food insecure adolescents (P = 0.003). Similarly, adolescents from households with severe food insecurity had lower mean grades mean (± SD) completed 5.5(± 2.7) compared to those from household that did not have severe food insecurity (6.2 ± 2.7, P < 0.001). The proportion adolescents who completed primary education (grade 8) was higher (31.5%) for food secure adolescents compared to food insecure adolescents (24.1%, P < 0.01).

Multivariable logistic regression model (Table 2) showed that food insecure adolescents were nearly twice as likely to be absent from school [OR 1.77 (1.34-2.33)] compared to their food secure peers after adjusting for age, gender, place of residence and gender of household head. Similarly, severe household food insecurity [OR 1.6 (1.27-2.06)], illness reported during the last month [OR 2.26 (1.68-3.06)] and the number of days the adolescents had to work for at least one hour per week [OR 1.16 (1.07-1.26)] were positively associated with school absenteeism. Adolescents who aspired to higher grades were less likely to be absent from school compared to their peers who aspired to a lower grade [OR 0.92 (0.88-0.96)].

**Table 2:** Predictors of school absenteeism in adolescents in Southwest Ethiopia

Predictors of school absenteeism	β	AOR^a ^[95% CI^b^]	P
Adolescent food insecurity			
Food insecure	0.57	1.77[1.34-2.33]	< 0.001
Food secure [reference]		1.00	
Illness within the last one month before the survey			
Yes	0.82	2.26[1.68-3.06]	< 0.001
No [reference]		1.00	
Severe household food insecurity			
Yes	0.48	1.62[1.27-2.06]	< 0.001
No [reference]		1.00	
The highest grade the adolescent aspires to complete	-0.08	0.92[0.88-0.96]	< 0.001
Number days in a week the adolescent has to work for least 1 hour	0.15	1.16[1.07-1.26]	< 0.001

^†^"Semi-urban" refers to small towns including Yebbu, Serbo and Dedo and "urban" refers to Jimma city.

Parameter estimates are adjusted for place of residence, gender of the adolescent, gender of the household head and the tabulated variables.

^a^Adjusted odds ratios as obtained from a multivariable logistic regression model.

^b^Confidence intervals, as obtained from a multivariable logistic regression model.

In bivariate linear regression models, adolescent food insecurity (P < 0.001), severe household food insecurity (P < 0.001), residence in rural area (P < 0.001) and female gender (P = 0.043) were negatively associated with educational attainment, while age of the adolescent (P < 0.001), highest grade aspired to be attained by the adolescent(P < 0.001), residence in urban area (P < 0.001), residence in semi urban area (P < 0.001) and household income (P < 0.001) were positively associated with educational attainment.

After adjusting for all other variables in the multivariable linear regression model (Table 3), food insecure adolescents attained a lower grade after 1 year of follow-up compared to food secure adolescents (β = -0.44, P < 0.001). Female gender (β = -0.18, P = 0.039), being a member of a severely food insecure household (β = -0.41, P < 0.001) and residing in a rural area (β = -2.70, P < 0.001) or semi-urban area (β = -0.92, P < 0.001) were negatively associated with educational attainment. However, age (β = 0.66, P < 0.001) and the highest grade aspired to be completed by the adolescent (β = 0.17, P < 0.001) were positively associated with educational attainment. The effect of household income and gender of the household head disappeared in the adjusted model.

**Table 3:** Predictors of highest grade completed§ by adolescents in Southwest Ethiopia

Predictors	Bivariate		Multivariable^¶^	
	β	P	β^∫^	P
		
Female gender	-0.21	0.083	-	-
Age in completed years	0.76	< 0.001	0.66	< 0.001
Adolescent food insecurity	-0.45	0.003	-0.44	< 0.001
Severe household food insecurity	-0.63	< 0.001	-0.41	< 0.001
The highest grade the adolescent aspires to complete	0.30	< 0.001	0.17	< 0.001
Residence in semi urban area*,^*f*^	0.70	< 0.001	-0.92	< 0.001
Residence in rural area^*f*^	-3.04	< 0.001	-2.70	< 0.001
Gender of the head of the household	0.77	< 0.001	-0.10	0.402
Household income	0.01	< 0.001	0.00	0.235

^§^The highest grade completed in school refers to the grade attained 1 year after the collection of data on predictors.

^∫^Coefficients as obtained from a multivariable linear regression model [adjusted R² = 0.48].

^¶^Multivariable models with the highest grade completed by the adolescents as dependent variable and predictors with P < 0.05 of the bivariate model.

*Yebbu, Serbo & Dedo Towns.

Parameters estimates adjusted for gender, household income, gender of the household head and the tabulated variables.

^*f*^Urban was used as a reference category in the multivariable model.

## Discussion

Food insecurity is a common problem among adolescents and households in Southwest Ethiopia [13,27]. The present study shows that food insecure adolescents and adolescents who were members of severely food insecure households were more likely to be absent from school and have a lower educational attainment in terms of the highest grade completed after 1 year of follow-up in Southwest Ethiopia. Food insecure adolescents had on average a lower grade completed (5.5) compared to food secure adolescents (6.0). The proportion of food insecure adolescents who completed primary education (24.1%) was also significantly lower than that of food secure adolescents (31.5%), although both proportions were lower than the national completion rate of primary schools in 2005-2006 [28]. Food insecure adolescents are likely to have a lower educational attainment due to several reasons including high absenteeism, illness, poor academic performance, academic delays, poor social functioning and behavioral problems [2,3,6]. According to Food and Agricultural Organization of the United Nations, hungry children start school later, drop out sooner and are also more likely to be absent and learn less while they do attend [29].

The effect of food insecurity on physical health and wellbeing is one of the ways through which food insecurity can erode educational attainment. As adolescence is a critical period of human growth and development, it can be affected by nutritional constraints resulting from food insecurity. There is an increasing recognition that ill health and malnutrition among school aged children have a major impact of on their cognitive development, learning and educational achievement. Improved health and nutrition are positively associated with enrolment at younger age, reduced absenteeism, less grade repetition and higher performance on test scores [30,31] due better cognitive development [32]. Cross-sectional studies from high income countries show that when food supplies are constrained at the household level, both adults and children use coping strategies such as reduction of meal size and meal frequency and eating lower quality foods [14], which in turn might induce malnutrition [8]. Similarly, cross-sectional [5] and longitudinal studies from high income countries [3,7] and a cross sectional study from a low income country [33] also showed that malnutrition is associated with poor cognitive development and decreased school performance in children. Malnourished children are less able to concentrate in school [3,18,29], which could lead to lower educational attainment.

In this study, adolescents who reported an illness during the last month were twice as likely to be absent from school. A report from the study area showed that food insecure adolescents had a higher frequency of illness than food secures ones [9,13]. Food insecurity not only jeopardizes the right to health but also has serious implications on education and schooling of adolescents. Secure access to food may enhance school attendance and overall health and well-being through decreasing use of negative coping strategies [30,31].

Food insecurity can also act as a psychological or emotional stress factor [15,34,35], affecting adolescent behavior and aspiration for further education. Our findings show that adolescents who aspire to complete higher grades were 18% less likely to be absent from school. The effect of food insecurity on children's well-being is related to its effect on the physical and socio-emotional aspects [3,4] that are linked to developmental consequences through nutritional and non-nutritional mechanisms [3]. While the nutritional effects might explain the negative consequences of inadequate nutrient intake on physical health and cognitive development, non-nutritional aspects of food insecurity relate to psychological effects including worry, anxiety or sadness about the family's food supply, feelings of having no choice in the foods eaten, shame/fear of being labeled as poor which are beyond the nutritional effects of food insecurity on adolescents [18]. Cook and Frank reported that food insecurity is a common risk to the growth, health, cognitive, and behavioural potential of children in constrained situations [35]. Children living in constrained environments were reported to have significantly higher cortisol levels due to stress [15]. Prolonged elevated cortisol level in humans have been associated with depression, cognitive deficits, and atrophy of brain structures involved in learning and memory [16,17] which may also lead to lower educational attainment. As a result of stress, anxiety and disrupted household dynamics [18], food-insufficient teenagers are more likely to have behavioral problems which makes them incompatible with school norms [5] negatively impacting on their educational attainment. Any of these mechanisms, alone or combined, could explain how food security affects the educational attainment of adolescents in the study area. Interventions focusing on improving the food insecurity situation such as conditional cash and/or food transfer programmes [36] need to consider these consequences on adolescents in identifying target groups.

After stratifying for gender, the bivariate analysis showed that in food insecure situations a higher proportion of girls are more likely to be absent from school compared to boys. This might be related to the social norms in the community that give low value female to education [37] that are potentially exacerbated when resources are constrained. A significantly larger proportion of girls was from food insecure households, food insecure themselves and reported an illness during the past one month compared to boys. This may be due to the selective buffering of boys from food insecurity by adult household members that is common in the study area [13]. The higher rates of food insecurity in girls could result in their school absenteeism through mechanisms discussed above. When entering gender as a variable in the multivariable model for school absenteeism however, no significant association was observed (results not shown), which indicates that the effect of gender is mediated by the other variables in the model.

School absenteeism in this study was also determined by a high frequency of work at the household level that the adolescent was engaged in. Adolescents who had to work longer hours per week were 1.4 times more likely to be absent from school. Children become absentees or dropout of school to help household labor as one of the coping strategies of food insecurity [38].

Our results imply that secure access of adolescents to food supply needs to be given attention to achieve the targets of the Millennium Development Goal. The findings provide arguments to incorporate food security in interventions that aim to address school attendance. Interventions including food stamp programs [2] and school breakfast programs [39] were reported to have beneficial effects for children on academic learning through improving dietary intake and/or reduction of stress. School supplementation program were also shown to have beneficial effects in reducing scholastic difficulties of adolescents [40]. In Ethiopia although such programs are being implemented by relief actors as part of an emergency response [41] there is no school feeding programme in non-emergency scenarios. There is a need to consider such interventions in potentially food constrained areas to prevent attrition of food insecure adolescents from school.

We acknowledge a number of limitations in our study. Although the adolescent and household food security scales were adapted from household food security scales after thorough discussion with the interview team who are residents of the study area, we might not rule out the possibility of some misclassification. The scales used assessed acute food insecurity as they covered only the last three months prior to the interview. The total survey period involved two seasons (rainy/peak hunger season) and spring season [42] which is better off in terms of food security and this matches a follow up period of one year. The proportion of food insecure households was 39.4% which was similar to estimations for urban areas of the region [38]. When interpreting the results of the study, it is important to note that our findings regarding food insecurity relate mainly to the study season and might not reflect the total year, but this is not a problem for the absenteeism variables. In addition, household food insecurity might be associated with adolescent food insecurity, which could introduce co-linearity in the models. The correlation coefficient between household food insecurity and adolescent food security was 0.2 and variance inflation factors in both logistic and linear regression models were generally low (the highest observed was 1.08), which indicates no considerable co-linearity in our analysis.

As the study involved adolescents who are at the different stages of academic status, we used the highest grade completed as a measure of educational attainment that can serve across all age groups. However, there are other measures of educational attainment that were not captured. The fact that we did not have data from the school records regarding the academic performances is also a limitation of our analysis.

## Conclusions

We report that food insecurity negatively impacts on school attendance and educational attainment of adolescents in the Southwest Ethiopia. The findings suggest that food security interventions should consider ways of enhancing school attendance and look into mechanisms for improving the diet of school children directly through school based programs.

## Competing interests

The authors declare that they have no competing interests.

## Authors' contributions

The authors' responsibilities were as follows: DL, CH, TB & AG: Designed and supervised the study and ensured quality of the data and made a substantial contribution to the local implementation of the study and PK, CL, CH & DL assisted in the analysis and interpretation of the data. All authors critically reviewed the manuscript. TB, the corresponding author did the analysis & drafted the manuscript and had the responsibility to submit the manuscript for publication.
